# Development and Transformation of Veterinary Experimental In Vitro Models: From 2D Culture to 3D Organoids

**DOI:** 10.3390/ani16030469

**Published:** 2026-02-03

**Authors:** Xuequan Hu, Yingying Xie, Jianfa Wang, Xingyun Zhang, Rui Wu

**Affiliations:** 1 College of Animal Science and Veterinary Medicine, Heilongjiang Bayi Agricultural University, Daqing 163319, China; 13836137830@163.com (X.H.);; 2 Qinghai Wildlife Rescue and Breeding Center, Xining 810000, China; 3 College of Biology and Agriculture, Jiamusi University, Jiamusi 154007, China

**Keywords:** veterinary medicine, organoids, 3D cell culture, animal welfare

## Abstract

In vitro models for animal experiments serve as a crucial bridge connecting basic research and clinical translation, and their developmental history profoundly reflects the paradigm shifts in life science research. This article’s narrative reviews the evolutionary path from traditional two-dimensional (2D) cell culture to advanced three-dimensional (3D) organoid technology, focusing on how organoid technology overcomes the limitations of traditional models in terms of physiological relevance, species specificity, and ethical constraints. The review article elaborates on the current state of organoid research in veterinary science, including the construction of models for organs such as the intestine, liver, and reproductive system in livestock and companion animals. Addressing existing technical bottlenecks—such as insufficient model complexity, lack of standardization, and difficulties in simulating vascularization and the immune microenvironment—future development directions are proposed, including multi-organ chips, AI-assisted analysis, and the integration of gene editing. Research indicates that with the deep integration of cutting-edge technologies such as biomaterials, microfluidics, 3D printing, and AI, organoid technology is progressively becoming a core driver for advancing veterinary precision medicine, holding broad application prospects.

## Simple Summary

Traditionally, animal cells are grown as flat, 2D layers in dishes for research, which fails to replicate their real 3D environment in the body. A breakthrough 3D technology called organoids—miniature, self-organized organ models grown from stem cells—is now transforming veterinary science. These organoids mimic the structure and partial function of real organs, providing a more realistic and ethical platform for studying diseases, testing drugs, and evaluating feed in livestock and pets. Models for intestines, liver, and other organs have been successfully established for species like cattle, pigs, dogs, and cats. While challenges remain in simulating features like blood vessels and immune cells, advances in combining organoids with gene editing and microfluidic chips are paving the way for more complex models. This progress will greatly advance precision veterinary medicine and support the “One Health” goal linking animal and human health.

## 1. Introduction

In vitro models for animal experiments serve as a critical bridge connecting basic research and clinical application, and their developmental trajectory profoundly mirrors the evolution of research paradigms in the life sciences. Each innovation in in vitro models, from initial simple 2D cell cultures to today’s complex 3D organoid systems, has significantly advanced our understanding of life phenomena and the elucidation of disease mechanisms. This transformation is particularly pronounced in the field of veterinary research. It not only provides an experimental platform that more closely mimics the in vivo environment for analyzing animal physiological and pathological processes but also opens new avenues for veterinary drug development. This review focuses on the technological evolution of in vitro models for animal experiments from 2D culture to 3D organoids and their application in veterinary research. It emphasizes how organoid technology overcomes the limitations of traditional models and provides an in-depth analysis of its unique value and application prospects. By delineating this developmental context, this review aims to clarify the profound impact of innovation in in vitro models on enhancing the level of veterinary research and advancing the development of veterinary precision medicine.

The literature was primarily retrieved from PubMed (last searched: 20 January 2026) using keywords including “3D cell culture”, “organoids”, and “veterinary research”. Priority was given to research articles published in the last five years. Given the limited volume of literature in this specific field, the criteria on publication year were applied flexibly to ensure coverage of key advancements.

## 2. The Development History of In Vitro Models

As a core tool in biomedical research, the development history of in vitro models profoundly reflects the continuous innovation in life science technologies. From the initial simple 2D monolayer cell culture to 3D culture systems capable of simulating the tissue microenvironment, and further to the emerging organoid technology in recent years, each technological breakthrough has significantly advanced the understanding of life phenomena and disease mechanisms. In the field of veterinary research, these models not only provide new perspectives for analyzing animal physiological and pathological processes but also lay an important foundation for veterinary drug development and disease model construction.

### 2.1. The Origin and Characteristics of 2D Cell Culture Technology

2D cell culture technology began with the pioneering work of Harrison in the early 20th century. Through subsequent optimization of culture media and standardization of vessels, it has become a cornerstone of biomedical research [1,2]. This technique involves cells growing in a monolayer on a flat substrate, offering advantages such as simple operation, low cost, ease of observation, and scalability, making it widely applicable in basic research, drug screening, and vaccine production [3,4].

However, the limitations of 2D models are also prominent. They fail to simulate the three-dimensional structure and extracellular matrix microenvironment in vivo, leading to significant differences in cell morphology, polarity, and gene expression compared to the actual in vivo situation [5,6]. Furthermore, 2D culture lacks key microenvironmental signals such as dynamic mechanical interactions between cells and the matrix, and nutrient gradients, which can easily lead to cell phenotype drift, affecting the reliability and reproducibility of experimental results [7,8].

### 2.2. The Emergence and Development of 3D Cell Culture Technology

To overcome the significant disparity between 2D cell culture models and the in vivo physiological environment, 3D cell culture technology emerged. 3D cell culture aims to simulate the three-dimensional spatial structure and microenvironment of cell growth in vivo, allowing cells to grow, proliferate, and differentiate under conditions closer to the physiological state, thereby better reproducing the biological characteristics and functions of tissues or organs [9,10]. Its core advantage lies in the ability to reconstitute cell-cell and cell-extracellular matrix (ECM) interactions, form more complex tissue structures, and generate gradients of nutrients, oxygen, and metabolites similar to those in vivo. These factors collectively regulate cell phenotype and functional state [7,11]. The development of 3D cell culture technology did not happen overnight but evolved from simple cell aggregates to complex biomimetic scaffold systems. Based on their technical principles, they can be broadly categorized into scaffold-based 3D culture systems and scaffold-free 3D culture systems.

#### 2.2.1. Scaffold-Free 3D Culture Systems

Cell self-assembly is the core mechanism of scaffold-free 3D culture, where cells spontaneously form multicellular aggregates with certain structures and functions through interactions mediated by their self-secreted ECM and cell adhesion molecules (such as cadherins). Spheroids are the most common scaffold-free 3D structures and can be obtained using methods such as hanging drop culture, low-adhesion plate culture, and rotating bioreactors [12,13]. Compared to traditional scaffold-based culture, spheroids exhibit higher cell density and tighter cell-cell connections, enabling them to mimic the structural characteristics of tumors, embryonic tissues, or certain glands. For instance, mesenchymal stem cell (MSC) spheroids formed under scaffold-free conditions demonstrate significantly superior anti-apoptotic capabilities, multi-directional differentiation potential, paracrine effects, and anti-inflammatory effects compared to MSCs cultured in 2D. This is likely related to the enhanced cell-cell contact, formation of gap junctions, and hypoxic microenvironment within the spheroids [14]. Furthermore, technologies such as microfluidics, magnetic levitation culture, and 3D bioprinting can also support scaffold-free 3D culture systems, offering possibilities for constructing highly biomimetic tissue models.

#### 2.2.2. Scaffold-Based 3D Culture Systems

Scaffold-based 3D culture systems introduce biocompatible materials as “scaffolds” for cell growth, providing cells with a three-dimensional network structure that mimics the ECM. This guides cells to colonize, migrate, proliferate, and differentiate within the scaffold, thereby forming three-dimensional models that more closely resemble in vivo tissue structures. Scaffold selection is central to this system; ideal scaffold materials should possess good biocompatibility, degradability, appropriate pore size and porosity, and physical and chemical characteristics similar to the target tissue [9,15,16].

Naturally derived scaffold materials were among the first to be widely used, including collagen, gelatin, and Matrigel™. Matrigel™ is a basement membrane matrix extracted from mouse Engelbreth-Holm-Swarm (EHS) sarcoma, rich in ECM components such as laminin and collagen IV. It can provide an excellent adhesion environment and bioactive signals for various cell types, promoting cell proliferation, differentiation, and the formation of tissue-like structures [17,18,19]. However, natural scaffold materials have drawbacks, including complex composition with significant batch-to-batch variation, potential introduction of pathogens or immunogenic substances, and difficulty in precisely controlling mechanical properties, which limit their potential for precise research and clinical applications [20,21]. To overcome the limitations of natural scaffolds, synthetic polymer material scaffolds have been gradually developed. These include oxidized alginate-gelatin, polylactic acid (PLA) and polyglycolic acid (PGA), whose chemical composition and physical properties can be precisely controlled through chemical synthesis methods, offering better reproducibility and batch-to-batch consistency [22,23].

### 2.3. Breakthrough Progress in Organoid Technology

Organoid technology represents one of the most transformative advances in three-dimensional (3D) cell culture in recent years and is often regarded as “miniature organs in a dish.” Specifically, organoids refer to 3D microtissues that form in vitro under defined 3D culture conditions—such as within extracellular matrix scaffolds (e.g., Matrigel)—through the self-organization and differentiation of stem cells, including adult stem cells (ASCs), embryonic stem cells (ESCs), or induced pluripotent stem cells (iPSCs). These microtissues possess structures and functions highly similar to their corresponding organs in vivo [1,24]. Compared to traditional 3D culture models, organoids not only contain multiple cell types but can also recapitulate the key structural features and partial physiological functions of the original organ. Therefore, they show immense application potential in modeling organ development, disease modeling, drug screening, and more.

#### 2.3.1. Core Characteristics of Organoids

Organoids typically exhibit the following core characteristics: Self-organization capability. The formation of organoids is not simply cell aggregation; rather, it is a process where stem cells or progenitor cells, regulated by specific signals, spontaneously form structures with polarity and tissue specificity through cell proliferation, migration, differentiation, and orderly arrangement. This process resembles the developmental pattern of organs in vivo [25,26]. Cellular complexity and tissue specificity: Organoids typically contain multiple cell types similar to those in the organ of origin and can form corresponding tissue architectures. Functional similarity: Organoids can, to a certain extent, simulate the physiological functions of the original organ and its responses to external stimuli. Potential for self-renewal and long-term culture: The stem cell component within organoids can continuously proliferate and replenish differentiated cells, allowing organoids to be stably passaged and maintained long-term in vitro [24,27].

These core characteristics make organoids closer to the in vivo reality compared to 2D cultures and traditional 3D models. Compared to animal models, organoids offer higher controllability, shorter experimental cycles, lower costs, and can avoid problems arising from interspecies differences. Especially under the “One Health” concept, using organoid models constructed from an animal’s own cells in veterinary research can more accurately reflect the physiological and pathological characteristics of that species [27,28].

#### 2.3.2. Stem Cell Sources and Directed Differentiation Techniques

The successful construction of organoids highly depends on suitable stem cell sources and their directed differentiation techniques. Currently, the stem cell sources for organoids mainly include the following categories: ASCs, ESCs and iPSCs.

ASCs were the earliest cell type used for culturing organoids, typically isolated from specific tissues such as the intestine, mammary gland, and hair follicle [28,29,30]. These stem cells are tissue-specific. Under culture conditions that add specific growth factors (e.g., EGF, Noggin, R-spondin) and inhibit differentiation signals, they can self-renew and differentiate into multiple cell types found in the tissue of origin, thereby forming the corresponding organoids. For example, cells isolated from the intestinal crypts of dogs or cats can be used to cultivate intestinal organoids with crypt-villus structures [28,31]. ASC-derived organoids have a stable genetic background and can retain individual and tissue characteristics, making them very suitable for disease modeling, drug screening, and personalized medicine research [27,32].

ESCs and iPSCs possess pluripotency, enabling the construction of organoids for various tissues and organs, and even complex organoid assemblies (assembloids) [33,34,35]. iPSCs are generated by introducing specific transcription factors (e.g., Oct4, Sox2) into somatic cells, reprogramming them back to a pluripotent state similar to ESCs [25,33]. The emergence of iPSCs overcomes the ethical and immune rejection limitations associated with ESCs, while retaining the pluripotency of ESCs. This makes it possible to use a patient’s (or animal’s) own cells to construct organoids with their genetic background, opening new avenues for researching genetic diseases and conducting personalized treatment assessments [33,34].

The directed differentiation technique for organoids is key to determining their structural and functional maturity. This process usually requires precisely simulating the signaling networks that regulate organ development in vivo. For instance, inducing brain organoids requires sequential activation or inhibition of signaling pathways such as Wnt, bone morphogenetic protein (BMP), and fibroblast growth factor (FGF) to mimic the formation of the neuroectoderm, neural tube closure, and brain region differentiation [36,37]. In recent years, by optimizing culture medium components, adjusting the combination and timing of growth factors, and introducing co-culture systems (e.g., with mesenchymal cells, endothelial cells), the differentiation efficiency and maturity of organoids have been significantly improved [38,39]. For example, in ovarian organoids, the interaction between vascular endothelial growth factor A (VEGF-A) and the Eph receptor B2 ligand-receptor pair can promote organoid development and maturation through cell communication between granulosa cells and oocytes [40]. Furthermore, the incorporation of endothelial cells promotes the structural maturation of nephrons, leads to the formation of fenestrated endothelium, and generates drug-responsive renin-expressing cells, all of which significantly enhance the physiological relevance and functionality of the organoid. Similarly, in lung organoids, the presence of endothelial cells improves barrier function, cellular diversity, and alveolar formation. Furthermore, after transplantation, these endothelial cells can integrate into the host circulation, further advancing the maturation process [41,42].

**Figure 1:**
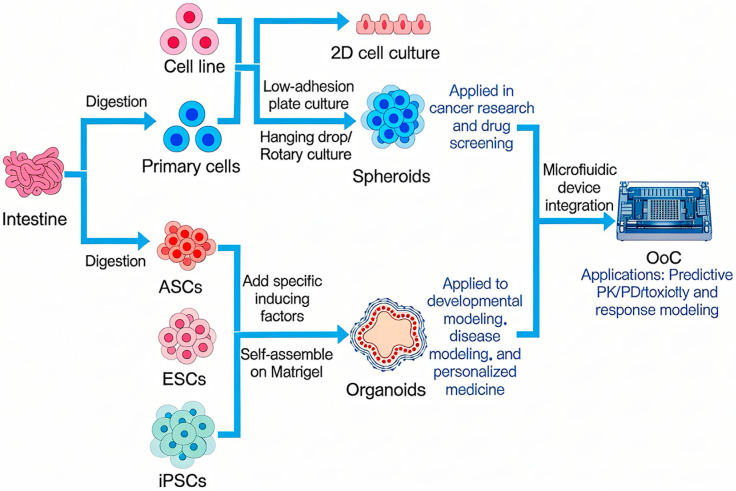
The Technological Spectrum and Integration of Advanced In Vitro Models.

To summarize and contrast these key methodologies, Figure 1 provides a schematic overview of the primary routes from cell sources to functional in vitro models and their translational applications.

## 3. Application of 3D Organoids in Veterinary Research

### 3.1. Construction and Characteristics of Organoid Models from Different Species

#### 3.1.1. Organoid Models from Traditional Livestock (Cattle, Pigs, Sheep)

The establishment of organoid models from traditional livestock such as cattle, pigs, and sheep represents a significant breakthrough in veterinary research in recent years. It provides novel tools for analyzing the physiological functions, disease mechanisms, and improving the breeding efficiency of these species. Compared to experimental animal models like rodents, livestock organoids better reflect species-specific biological characteristics, offering irreplaceable advantages, especially in the study of zoonotic diseases [43]. As important agricultural animals and large animal models for biomedical research, the construction and application of intestinal organoids from cattle and pigs have garnered particular attention.

Regarding bovine organoid models, researchers have successfully isolated intestinal crypts from adult bovine small intestine tissue and established a long-term, stable, and passagable bovine intestinal organoid culture system [44]. These organoids not only exhibit the typical crypt-villus structure morphologically but also express intestinal stem cell markers (e.g., Lgr5), differentiated cell markers (e.g., MUC2 in goblet cells and Chromogranin A in enteroendocrine cells), and possess barrier function and substance transport capabilities similar to those in vivo [44]. Furthermore, the construction of bovine oviduct organoids provides a platform for studying the early embryonic development microenvironment in cattle and improving the efficiency of in vitro fertilization [45,46].

Similarly, significant achievements have been made in porcine organoid models. Due to the closer similarity of porcine intestinal structure and physiological function to humans, porcine intestinal organoids serve not only as tools for veterinary research but are also widely used in comparative medical studies. Researchers have isolated crypts from porcine jejunum and ileum tissues and successfully constructed porcine intestinal organoids by culturing them in medium containing growth factors such as Wnt3a, R-spondin 1, and Noggin [47,48]. These organoids can mimic the physiological structure of the porcine intestine, including the villus epithelium, goblet cells, and Paneth cells, and maintain self-renewal capacity during long-term culture.

#### 3.1.2. Organoid Models of Companion Animals (Dogs, Cats)

As important companions to humans, the health and welfare of companion animals (dogs, cats) are receiving increasing attention. Dogs and cats not only suffer from many complex diseases similar to humans (such as cancer, inflammatory bowel disease, neurodegenerative diseases), but their spontaneous disease models also hold extremely high value for translational medical research [49]. Therefore, the construction and application of canine and feline organoid models have become a hot topic in veterinary research.

Research on canine organoid models has covered multiple organ systems, with intestinal and hepatic organoids being the most well-established [49]. Researchers have successfully isolated intestinal stem cells from jejunal tissues and endoscopic biopsy samples of both healthy dogs and dogs suffering from gastrointestinal diseases [such as Inflammatory Bowel Disease (IBD) and intestinal cancer], establishing stable canine intestinal organoid (enteroid and colonoid) culture systems [50]. These organoids can be passaged long-term while maintaining the intestinal crypt-villus structure, and express typical intestinal stem cell markers (e.g., Lgr5, Olfm4), absorptive cell markers (e.g., SI), goblet cell markers (e.g., MUC2), and enteroendocrine cell markers (e.g., CGA) [51].

Although the development of feline organoid models started slightly later, significant progress has been made in recent years, particularly regarding reproductive and ocular tissues. Researchers successfully established the first feline oviduct organoid model. By optimizing the culture medium components (e.g., adding WNT agonists, TGFβ inhibitors), the isolated oviduct epithelial cells self-assembled into organoids with lumen structures, expressing oviduct epithelial cell markers (e.g., CK18, E-cadherin) [52,53]. Furthermore, the successful culture of feline corneal epithelial organoids has been achieved; these organoids express corneal epithelial stem cell markers (e.g., p63) and differentiation markers (e.g., AQP1) [54].

The establishment and standardization of canine and feline organoid models not only provide precise disease models for veterinary clinical practice but also contribute to comparative medicine and translational research due to the similarity of their diseases to human conditions. For instance, canine IBD organoid models can aid in understanding the complex pathogenesis of human IBD [50]; feline intestinal organoid models infected with coronavirus provide references for studying human coronaviruses (e.g., SARS-CoV-2) [55]. However, current canine and feline organoid models still face challenges such as high culture costs, the need for improved long-term culture stability, and the lack of microenvironmental components like blood vessels and immune cells. These issues need to be addressed in the future through innovations in biomaterials (e.g., synthetic hydrogels replacing Matrigel) and co-culture techniques [56,57].

#### 3.1.3. Organoid Models of Laboratory Animals

Organoid models of laboratory animals (primarily rodents) are among the earliest established and most widely used in biomedical research. They provide powerful tools for understanding fundamental biological processes and disease mechanisms, and offer technical paradigms for constructing organoid models of large animals and humans. Wildlife organoid models represent an emerging field with immeasurable value for conservation biology, wildlife disease prevention and control, and research into the origins of zoonotic diseases.

Organoid models for various organs—such as intestine, brain, liver, kidney, and lung—have been most successfully established in the mouse, making them gold-standard systems in biomedical research [58]. Although this review focuses on veterinary research, the accumulated technical expertise and research findings from mouse organoid models provide crucial references for constructing organoids from other animal species [59]. For instance, the culture system for mouse intestinal organoids (relying on the regulation of signaling pathways like Wnt, Notch, and BMP) has been widely adapted and modified for application in cultivating intestinal organoids from livestock like cattle and pigs, and companion animals like dogs [43,51]. The advantages of mouse organoid models lie in their well-defined genetic background and ease of genetic editing, enabling the rapid construction of various genetically engineered organoid models to study the role of specific genes in disease pathogenesis and progression [60]. However, physiological and pathological differences between mice and humans or other animals limit the value of direct extrapolation, necessitating studies using organoid models from the target species.

Rat organoid models offer advantages in certain areas (such as metabolic studies and neurodegenerative diseases) that mouse models cannot match. Rats are larger in size, and their organ structures are closer to those of humans. The successful establishment of rat organoid models, such as intestinal and brain organoids, provides more refined in vitro alternatives for toxicology testing and disease modeling involving rats in veterinary research [61]. Furthermore, organoid models for other laboratory animals like guinea pigs and rabbits are gradually being developed, enriching the options for laboratory animal models.

### 3.2. Drug Screening and Toxicity Assessment

#### 3.2.1. Efficacy and Toxicity Evaluation of Conventional Drugs

In veterinary medicine, 3D organoid technology is increasingly replacing or complementing traditional 2D cell culture and animal testing for the evaluation of conventional drug efficacy and toxicity. It has emerged as a novel in vitro model offering greater physiological relevance, reproducibility, and predictive capability. Compared to 2D cultures, organoids better mimic the structure and function of in vivo tissues. This includes capturing cellular heterogeneity, cell-cell communication, extracellular matrix interactions, and metabolic activity, thereby providing a more accurate reflection of a drug’s in vivo absorption, distribution, metabolism, excretion processes, and potential toxic side effects. Compared to animal experiments, organoid models not only reduce the use of research animals—aligning with the 3R principles—but also shorten experimental timelines, lower costs, and circumvent biases in efficacy and toxicity assessment arising from interspecies differences [43,62].

For evaluating the efficacy of veterinary drugs, intestinal and liver organoids are the most extensively utilized. For example, when assessing anti-infective drugs for intestinal pathogens, porcine or bovine intestinal organoids can be co-cultured with relevant pathogens to establish infection models. The therapeutic efficacy of a drug can then be evaluated by measuring its inhibition rate on pathogen replication, its ability to ameliorate pathological damage in the organoids, and its modulatory effects on host defense gene expression [43,63]. Such models allow for the in vitro simulation of drug-pathogen-host intestinal epithelium interactions, offering superior predictivity of in vivo efficacy compared to traditional 2D cell infection models. The liver is the primary site for drug metabolism and a major target for drug-induced toxicity. Liver organoid models derived from dogs, pigs, and cattle have been successfully employed to assess drug hepatotoxicity and metabolic stability [49]. Parameters such as organoid viability, lactate dehydrogenase release, apoptosis rate, and levels of specific liver injury markers measured after drug treatment can effectively evaluate potential liver toxicity [27,56]. Cats exhibit unique sensitivities to certain drugs (e.g., acetaminophen). Utilizing feline liver organoids enables in-depth study of the metabolic characteristics and toxicity mechanisms of these drugs in cats [52,53].

#### 3.2.2. Application in Veterinary Vaccine Development

3D organoid technology provides a novel and efficient in vitro research platform for veterinary vaccine development, demonstrating unique advantages across antigen screening, vaccine potency evaluation, and investigation of immune mechanisms. Compared to traditional vaccine development heavily reliant on animal experimentation, organoid models can simulate the microenvironment and cellular composition of host target organs in vitro. This allows for a more accurate reflection of in vivo processes such as vaccine antigen presentation, immune cell activation, and the induction of protective immune responses. Consequently, this approach accelerates the screening and optimization of vaccine candidates, reduces development costs and timelines, and decreases the use of experimental animals [64].

In the area of vaccine antigen screening and optimization, organoid models can simulate the natural infection route of pathogens and the cell types of target organs, thereby enabling more effective identification of key antigens capable of inducing protective immunity. For instance, porcine intestinal organoids can serve as a platform for antigen presentation and immunogenicity evaluation for enteric coronaviruses like Porcine Transmissible Gastroenteritis Virus (TGEV) [28,65]. By loading different viral proteins or virus-like particles onto intestinal organoids, one can assess the expression efficiency, processing, and presentation capabilities of the antigens within intestinal epithelial cells, as well as their impact on the organoid’s own structure and function. Preliminary judgment on the antigen’s immunogenicity can be made by detecting the expression of immune-related genes and the secretion of relevant cytokines within the organoids following antigen stimulation [65]. This organoid-based antigen screening model offers better prediction of in vivo immunogenicity compared to traditional 2D cell line expression systems, as it incorporates a cellular differentiation state and antigen presentation microenvironment closer to the natural condition.

For vaccine potency evaluation, organoids can be combined with corresponding pathogen infection models to establish an in vitro simulation system of “vaccine immunization-pathogen challenge,” used to assess the anti-infective protective efficacy induced by the vaccine. For example, using a porcine intestinal organoid infection model, organoids are first co-incubated with a candidate vaccine to simulate the vaccination process, followed by a challenge with a virulent strain of TGEV or *Salmonella*. The protective efficacy of the vaccine is then evaluated by measuring pathogen replication titers within the organoids, the extent of organoid pathological damage, and the activation status of host defense mechanisms [43,65]. For vaccines whose protection is mediated by cellular immunity, organoids can be co-cultured with immune cells to construct an organoid-immune cell co-culture model. This model is used to assess vaccine-induced specific T-cell proliferation, cytotoxic effects, and antibody secretion [66,67].

In research on vaccine immune mechanisms, organoid models provide a refined tool for elucidating the mechanisms of vaccine-induced local immune responses. For example, single-cell RNA sequencing of organoids treated with a vaccine can analyze gene expression changes in different cell subsets during the immunization process, identifying key cell types and molecular pathways involved in antigen presentation, immune activation, or immunoregulation [68,69].

In summary, the construction of organoid models from traditional livestock overcomes the limitations of traditional 2D cell culture and animal experiments. They can maintain tissue-specific structures and functions long-term in vitro, providing efficient and ethical alternatives for species-specific physiological studies, disease modeling, and drug screening. They also build a bridge for cross-species research under the One Health concept [66].

As detailed in Table 1, organoids have been successfully established from a wide range of organs—including intestine, lung, brain, and reproductive tissues—in key species such as bovines, porcine, canines, and felines, enabling research into development, infection, cancer, and toxicology.

As the field advances, future work should transition to standardized, criteria-driven comparisons. Key benchmarks should include functional maturity, validated readouts, reproducibility, and cost-effectiveness across species. Establishing such a framework will enable researchers to critically select the most suitable models for specific translational applications.

## 4. Challenges and Prospects of In Vitro Models and Organoid Technology

### 4.1. Technical Bottlenecks and Standardization Issues

#### 4.1.1. Inadequate Model Maturity

Although 3D organoid technology shows great potential in veterinary research, the insufficient complexity and maturity of the models remain a core bottleneck limiting their widespread application. While current organoid models can partially simulate the structure and function of organs in vivo—for instance, bovine intestinal organoids can recapitulate the intestinal crypt structure and key absorptive functions [68], and canine liver and intestinal organoids can express specific tissue markers [49]—significant gaps remain compared to real organs in terms of cellular heterogeneity, organizational structural complexity, and the completeness of physiological functions [66,75]. For example, in infectious disease research, epithelial-only organoids struggle to replicate the dynamic interactions between pathogens and the host immune system, greatly limiting the elucidation of the complete disease pathogenesis [76]. Furthermore, the inadequate maturity of organoids is a widespread issue. Organoids cultured in vitro often remain at a relatively immature developmental stage, and their physiological functions and gene expression profiles differ from those of the corresponding organs in adult animals. This immaturity may lead to biased results in drug toxicity assessment and metabolism studies, affecting their reliability as preclinical models [72,73].

#### 4.1.2. Standardization and Reproducibility of Culture Systems

The standardization of culture systems and the reproducibility of experimental results are key hurdles for the transition of organoid technology from laboratory research to industrialization and clinical application. This issue is particularly pronounced in veterinary research due to the involvement of multiple species and tissue types. Currently, significant variations exist in veterinary organoid culture methods across different laboratories, including cell isolation protocols, culture medium composition and growth factor combinations. These differences directly lead to inconsistencies in organoid model quality and make experimental results difficult to compare [74,77].

Optimizing and standardizing culture medium components is central to improving reproducibility. Most veterinary organoid cultures still adapt formulations from human or mouse studies, lacking dedicated media tailored to specific animal species or tissue types. The type and concentration of growth factors are crucial for organoid differentiation and maintenance, yet responses to growth factors may vary between species [75]. Furthermore, the use of serum or other animal-derived components not only introduces batch-to-batch variation but also carries the risk of pathogen contamination, affecting experimental stability and safety [78].

Standardizing cell sources and isolation methods is also crucial for ensuring reproducibility. Veterinary organoids are derived from diverse cell sources, including embryonic stem cells, induced pluripotent stem cells (iPSCs), and adult tissue stem cells, each with different differentiation potentials and proliferation characteristics [72,75]. Even for stem cells from the same tissue, slight variations in isolation and purification methods (such as enzymatic digestion time, centrifugation speed) can lead to significant differences in organoid-forming capacity [68]. Establishing unified cell identification and quality control standards is essential for ensuring the quality of the starting cells [28,49].

#### 4.1.3. Challenges of Vascularization and Innervation

Vascularization and innervation are major obstacles limiting the application of organoids in regenerative medicine and complex disease modeling. In vivo, the vascular system not only supplies oxygen and nutrients to tissues but also participates in cell signaling and the clearance of metabolic waste products. Innervation, on the other hand, regulates the functional activity and repair processes of organs. However, most existing veterinary organoid models lack functional vascular networks and nerve fibers, which severely restricts their potential for size growth, functional maturation, and integration with a host [79].

Nutrient and oxygen supply within organoids primarily relies on diffusion, which typically limits the diameter of organoids to a few hundred micrometers. When organoid volume increases, the central region is prone to hypoxia and necrosis, affecting their long-term culture and functional maintenance [80]. To address this issue, researchers have attempted various vascularization strategies. One method involves co-culturing organoids with vascular endothelial cells, inducing the endothelial cells to migrate into the organoid and form vessel-like structures. Another promising approach utilizes microfluidic technology (organ-on-a-chip) to construct dynamic perfusion systems that promote organoid vascularization and maturation by simulating the in vivo hemodynamic environment. Organ-on-a-chip platforms can provide precise fluid shear stress and nutrient exchange and have been shown to significantly increase the vascular density and function of organoids [81,82]. Simulating innervation is even more complex than vascularization. Currently, research on innervation in veterinary organoids is virtually non-existent, with only a few studies exploring interactions between neural organoids and other organoids in human and mouse models.

#### 4.1.4. Difficulties in Simulating the Immune Microenvironment

The immune system is a crucial barrier for animals to defend against pathogen invasion and maintain internal homeostasis. Its interactions with organs and tissues are vital in both health and disease states. However, simulating the immune microenvironment is a significant weakness in current organoid models, greatly limiting their application in areas such as infectious diseases, cancer immunology, and vaccine development [83]. Veterinary organoid models typically contain only parenchymal cells (e.g., epithelial cells) and lack immune cells as well as the associated network of cytokines and chemokines, making it difficult to recapitulate the complex immune response processes occurring in vivo [76].

A primary challenge in simulating the immune microenvironment is how to effectively integrate immune cells into the organoid model. Two main strategies currently exist: first, co-culturing immune cells derived from peripheral blood or spleen with organoids; second, differentiating induced pluripotent stem cells into specific immune cell types and then assembling them with organoids [84,85]. In the veterinary field, similar attempts are just beginning, such as co-culturing mouse alveolar macrophages with lung organoids to simulate the immune response following influenza virus infection [70,76]. Furthermore, organoid models provide a new platform for studying viral, bacterial, and parasitic infections, the lack of immune cells prevents the complete simulation of the inflammatory response and immune clearance processes post-infection [68,71].

### 4.2. Future Development Directions and Prospects

#### 4.2.1. Multi-Organ Chips and Organoid Assembloids

Multi-organ chips (Organ-on-a-Chip, OoC) and organoid assembloid technologies represent the cutting edge of in vitro model development. By integrating functional units of multiple organs or tissues, they simulate complex physiological and pathological processes in vivo more comprehensively, providing a research platform for veterinary studies that more closely resembles the real internal environment. While traditional single-organoid models can simulate the structure and function of specific organs, they cannot replicate the interactions between organs or systemic regulatory mechanisms, such as the absorption, distribution, metabolism, and excretion (ADME) of drugs in vivo, or the multi-organ involvement in systemic diseases [86].

#### 4.2.2. Application of Artificial Intelligence and Machine Learning in Organoid Data Analysis

The rapid development of artificial intelligence (AI) and machine learning (ML) technologies provides powerful tools for analyzing the massive datasets generated in organoid research, promising revolutionary breakthroughs in organoid construction optimization, functional prediction, disease modeling, and drug screening. Organoid systems, especially complex models incorporating microfluidics, bioprinting, and multi-omics analyses, generate multidimensional big data encompassing morphology, gene expression, proteomics, metabolomics, and electrophysiological activity [87,88]. Traditional data analysis methods struggle to process and interpret these data efficiently. In contrast, AI/ML algorithms can delve into the biological patterns embedded within the data through pattern recognition, feature extraction, and predictive modeling, thereby accelerating the development and application of organoid technology [88].

#### 4.2.3. Potential of Combining Gene Editing with Organoids in Veterinary Precision Medicine

Gene editing technologies, particularly the advent of the CRISPR-Cas9 system, provide an unprecedented tool for precise manipulation of the genome in in vitro models. The combination of gene editing with organoid technology has given rise to “gene-edited organoid” models. This integrated approach shows great potential in veterinary precision medicine, promising to open new avenues in disease modeling, gene function research, livestock breeding improvement, and personalized treatment. Organoids serve as an ideal vector for gene editing, capable of simulating the physiological environment of tissues in vitro, allowing functional validation of edited cells within a three-dimensional structure. Conversely, gene editing equips organoid models with the ability to study specific gene functions and model genetic diseases. The combination of these two technologies significantly expands the depth and breadth of veterinary research.

## 5. Conclusions

This review provides a comprehensive overview of veterinary organoid technologies, consolidating current methodologies and applications. As reflected herein, animal experimental in vitro models have undergone a remarkable evolution from traditional 2D culture systems to 3D organoid systems. This technological transformation has provided veterinary research with experimental platforms that more closely mimic physiological conditions. Organoid technology can simulate organ structure, function, and species specificity. Leveraging this ability, researchers have successfully established organoid models for multiple organs (e.g., intestines, liver, reproductive systems) in traditional livestock, companion animals, and laboratory animals. It has found broad applications in disease modeling, drug screening and toxicity assessment, and vaccine development, among other fields.

Despite these advances, the application of organoid technology in veterinary science still faces several technical bottlenecks, such as insufficient model maturity, challenges in standardizing culture systems, and the lack of vascularization and immune microenvironment simulation. Looking ahead, further integration of interdisciplinary approaches—including multi-organ chip systems, artificial intelligence-assisted analysis, gene editing technologies, as well as biomaterials and 3D printing—is expected to drive organoid models toward greater complexity, standardization, and functional integrity.

Positioned as a foundational resource, this work aims to support the next essential phase: the systematic, criteria-based comparison of these models. Overall, organoid technology is gradually becoming an essential tool in veterinary precision medicine. It not only holds the potential to reduce the use of experimental animals and enhance research reproducibility, but also opens new pathways for deepening our understanding of animal disease mechanisms and accelerating the development and translation of veterinary drugs. With ongoing optimization and innovation in related technologies, organoids are poised to play an increasingly significant role in both veterinary research and clinical practice.

## Figures and Tables

**Table 1:** Summary of Organoid Models Across Species.

Animal Species	Organ/Tissue Type	Summary of Research Core Keywords
Mouse	Brain	Validation of brain ribosomopathy model [60]
	Intestine	Injury regeneration mechanisms [58]
	Lung	Lung organoid-macrophage co-culture [70]
	Reproductive System	Acr-Luc knock-in model, real-time imaging for reproductive toxicity [62]
Bovine (Cow)	Intestine	Infection models [43], long-term culture [44], functional validation [68]
	Oviduct	Hormonal response [45], heat stress response [46], multi-species comparison [52]
Porcine (Pig)	Intestine	PEDV infection [47], developmental transition [48], TGEV infection [65], DON toxicity [69]
	Lung/Airway	Airway organoids, PRCoV infection, innate immunity [71]
	Nervous System	Dopaminergic neurons, midbrain organoids [72]
Canine (Dog)	Intestine	Barrier integrity [31], standardized culture [49], IBD proteomics phenotype [50], translational research [51], drug permeability [63]
	Liver	Standardized organoid culture [49]
	Cornea	Corneal organoids, disease modeling [54]
	Tumors	Metabolic analysis [73], mesothelioma organoids [74]
Feline (Cat)	Intestine	Feline coronavirus, infection model [55]
	Oviduct	Oviductal organoids, cryopreservation [53]
	Cornea	Corneal organoid culture [54]

## Data Availability

No new data were created or analyzed in this study. Data sharing is not applicable to this article.
